# The Role of Intimate Partner Violence, Couple Dissatisfaction and Parenting Behaviors in Understanding Parental Burnout

**DOI:** 10.1007/s10826-021-02218-5

**Published:** 2022-01-15

**Authors:** Katharina Prandstetter, Hugh Murphy, Heather M. Foran

**Affiliations:** Institute of Psychology, Department of Health Psychology, University of Klagenfurt, Klagenfurt, Austria

**Keywords:** Parental burnout, Dysfunctional parenting, Intimate partner violence, Couple dissatisfaction

## Abstract

Parental burnout (PB), a relatively new and under-studied construct, is defined as a condition resulting from chronic parenting stress. While recent research confirmed its negative associations with familial variables, such as relationship satisfaction and positive parenting practices, little is known about the role of intimate partner violence (IPV) and how it relates to parental burnout. The present study, therefore, aimed to extend existing knowledge on chronic parenting stress by 1) testing for the mediational role of couple dissatisfaction in explaining the link from IPV victimization to PB as well as the link from IPV victimization to dysfunctional parenting, and 2) investigating how specialist gender roles and parental responsibilities for child care relate to IPV victimization and PB. Data collection was part of an international collaboration on factors related to parental satisfaction and exhaustion across different countries. Self-report data from Austrian mothers (*N* = 121) were collected online and analyzed using structural equation modeling. Results indicated that couple dissatisfaction mediates the link from IPV victimization to PB, as well as IPV victimization to dysfunctional parenting. Furthermore, only specialist gender roles were significantly related to IPV, while parental responsibilities for child care did not significantly relate to experiences of violence. Additionally, neither specialist gender roles nor parental responsibilities were significantly associated with PB in the final model. Overall, our findings connect to family models, such as the Family System Theory and Spillover Theory, underscoring the importance of couples’ relationship quality for understanding parental burnout and parenting behaviors in mothers.

## Highlights

- Couple dissatisfaction mediates the link from couple violence to parental burnout
- Couple dissatisfaction helps explain the link from couple violence to parenting
- Specialist gender roles relate to experiences of physical violence in mothers

Perceiving stress from time to time when raising children is a common experience for both fathers and mothers (Crnic, 2002). Minor hassles are part of every parent’s daily routine, independent of their social background and context. However, once stress becomes chronic, this can have detrimental effects on both parents’ and child(ren)’s mental health (Crnic et al., 2005, Huth-Bocks & Hughes, 2008). Parental burnout (PB) is a rather new phenomenon, and it describes a syndrome developing in response to chronic parenting stress (Mikolajczak et al., 2019). Three dimensions characterize PB: overwhelming exhaustion (being a parent requires too much of the available resources), emotional distancing (a reduced parent-child interaction), and a sense of ineffectiveness (believing you cannot manage effectively as a parent) (Le Vigouroux & Scola, 2018, Mikolajczak & Roskam, 2018). It can affect fathers as well as mothers and, depending on the sample, the prevalence reported is between 8% and 36% (Lindström et al., 2011, Roskam et al., 2017). Research suggests that PB arises when there is a chronic imbalance between factors which increase parental stress (i.e., risks/demands) and factors which decrease parental stress (i.e., access to resources) (Mikolajczak & Roskam, 2018). However, despite its similarity to job burnout, depression, and ‘normative’ parenting stress, PB has shown to be theoretically and empirically distinct from the aforementioned constructs (Mikolajczak et al., 2019, Roskam et al., 2017).

Since the emergence of research on PB, several studies have confirmed its negative associations with parental mental health outcomes, such as suicidal ideation, anxiety, and depression (Lebert-Charron et al., 2018, Mikolajczak et al., 2019). A large body of research has also highlighted the importance of parent-child interactions, as parental stress and experiences of PB have both been identified as risk factors for child neglect and violence (Lebert-Charron et al., 2018, Mikolajczak et al., 2019, Stith et al., 2009). Given the harmful effects of chronic parenting stress on the parents as well as children, much effort has been made to identify risk and protective factors related to PB. For instance, repetitive exposure to parental demands reflected in high levels of responsibility for parental chores and duties as well as perceived lack of support by the partner, family, or nurseries may increase the risk of parents developing PB (Mikolajczak & Roskam, 2018). Additionally, norms prescribing women to be ‘ideal’ parents are also assumed to be stress-inducing (e.g., pressure to be a perfect mother, trying to avoid “mistakes”), thereby making mothers more vulnerable to PB (Douglas & Michaels, 2005). As to potential protective factors, past research showed that parents who display greater emotional stability, parental self-efficacy and who engage in positive parenting practices are less likely to be affected by PB (Mikolajczak, Raes, et al., 2018). Furthermore, good co-parenting and communication skills, as well as higher levels of couple satisfaction, are assumed to reduce the risk of parental burnout (Lindström et al., 2011, Mikolajczak, Raes, et al., 2018).

## Intimate Partner Violence

Despite several already established associations between PB and family-related variables, such as relationship satisfaction and parenting practices (Mikolajczak, Raes, et al., 2018), less is known about the role of intimate partner violence (IPV) and its associations with PB. According to Heyman et al. (2013), IPV can be defined as any physical, verbal/symbolic, or sexual acts that can cause or have reasonable potential to cause harm to an intimate partner. It involves three different types: sexual, physical, and emotional violence, which often co-occur (Foran & Phelps, 2013). The severity of IPV can vary, ranging from less deleterious behavior, such as grabbing, to more harmful acts, including kicking or stabbing (Slep et al., 2015). Research has shown that relationships with IPV compared to those without are more likely to be characterized by couple conflict and dissatisfaction (Capaldi et al., 2012, Stith et al., 2004).

Relationship violence is a global phenomenon with adverse effects on the individual, family, and societal level (Garcia-Moreno et al., 2006, Pallitto & Carcia-Moreno, 2013). Worldwide, 30% of women are affected by physical or sexual IPV (Garcia-Moreno et al., 2013). In Europe alone, 25% of women reported having been a victim of physical or sexual IPV at least once in their life (World Health Organisation, 2013). Data from the violence against women EU-wide survey indicated that 13% of Austrian women reported having been a victim of IPV (European Union Agency for Fundamental Rights, 2015). As past research highlighted, IPV has become a serious public health concern, with numerous theories being established to explain its emergence (Ali & Naylor, 2013).

### Effects of IPV on Parental and Child Level

Not only PB but also IPV comes along with several negative mental health outcomes, including depression, anxiety, drug abuse, and self-harm (Ehrensaft et al., 2006). A review on the consequences of IPV also highlighted its links to a number of physical health problems, such as chronic pain disorders, psychosomatic health complaints as well as lower levels of overall physical health (Dillon et al., 2013). IPV, however, is also presumed to exert influences beyond the couple level, especially once children are involved. Following Family System Theory (FST; Cox & Paley, 1997), individuals within a family are interdependent, and due to their spatial and private proximity, the behaviors and emotions of family members are intertwined. One approach that has highlighted associations between interparental relationship quality and parent-child interaction is the Spillover Theory (Engfer, 1988, Erel & Burman, 1995), suggesting that negative emotions deriving from interparental relationship conflict directly “spillover” to the parent-child relationship (Jouriles et al., 2016, Krishnakumar & Buehler, 2000). This theory has found empirical support in several studies (Gerard et al., 2006, Sherrill et al., 2017, Stroud et al., 2015), emphasizing the relevance of interparental conflict not only for children’s mental health, such as externalizing and internalizing disorders, but also physical health, such as sleeping and eating disorders (Artz et al., 2010, Huth-Bocks & Hughes, 2008).

### Gender Roles and Intimate Partner Violence

The feminist movement was among the first to campaign and raise awareness of violence against women as well as the need for the establishment of protection services (McPhail et al., 2007). Feminist perspectives argue that traditional sex roles and power imbalances constitute risk factors for male to female violence and that aggression perpetrated by males serves as a way to exert control over their partners (Ali & Naylor, 2013). According to this theory, social inequality manifests in traditional roles assigned to women and men, such that women are held responsible for child nurturing and caretaking, while men are in charge of authority and decision-making (Yllö & Bograd, 1988). Feminist theories have also found empirical support in several studies identifying traditional sex-role ideologies as risk factors for physical and psychological violence, with women more often being victims of physical and sexual violence than men (Schumacher et al., 2001, Stith et al., 2004). Although past research has emphasized the negative role of power imbalances and physical violence in low-income settings, IPV is also prevalent in high-income countries, such as Austria, underscoring its relevance on a global level (Gracia & Merlo, 2016).

## Aim of the Current Study

In keeping with FST (Cox & Paley, 1997), and Spillover Theory (Erel & Burman, 1995) proposing that interparental conflict produces feelings of anger and frustration, which then shape negative parenting behaviors, the main goal of this study was to test the role of couple dissatisfaction as a mediator of the relation between IPV victimization to PB and the relation between IPV victimization to dysfunctional parenting strategies. To the best of our knowledge, no recent study has investigated how IPV contributes to PB and how this association is related to other family-related variables, such as couple dissatisfaction and parenting practices, which have been linked to both IPV and PB in past research (Greeson et al., 2014, Mikolajczak, Raes, et al., 2018). As IPV has shown to be associated with relationship dissatisfaction (Shortt et al., 2010, Testa & Leonard, 2001), which in turn has been linked to negative parenting and parenting stress (Erel & Burman, 1995, Gao et al., 2019), we assumed that IPV alone will not fully account for increases in feelings of PB and the use of dysfunctional parenting behaviors, but instead may be better understood by an interplay of variables on the parent-child and relationship level (Stith et al., 2008).

Secondly, we also expected that specialist gender roles (mothers are responsible for children and family; fathers are in charge of earning money) will be linked to more responsibilities for child(ren)’s basic needs (i.e., feeding and hygiene) in mothers. This is in line with research indicating that partners’ traditional views on ‘appropriate’ roles for parents will eventually shape their behaviors, resulting in fathers being in charge of financial aspects and mothers being responsible for childcare, family, and household (Thompson & Walker, 1989). As a recent report from the Austrian Presidency of the Council of the European Union (2018) revealed, child care supervision responsibilities have also remained a major reason for women working part-time.

Following feminist theories, we further assumed that specialist gender roles and responsibility for child(ren)’s basic needs will be associated with IPV victimization in mothers. This is also in line with research emphasizing links between womens’ physical victimization and gender roles (Herrero et al., 2017), as well as studies showing that being a female victim is related to more harmful physical and psychological consequences (Caldwell et al., 2012).

Lastly, as positive attitudes towards specialist gender roles are assumed to reflect parental role division within the family, our final aim was to investigate whether greater maternal responsibility for child care and positive attitudes towards specialist gender roles are linked to PB. In keeping with the concept of balance and demands, we expected that positive attitudes towards specialist gender roles and greater parental responsibility will make mothers more vulnerable to chronic parenting stress. Additionally, we expected that being a victim of IPV will put mothers at even higher risk of developing PB and the use of dysfunctional parenting behaviors, as interparental conflict and relationship dissatisfaction might additionally decrease resources to cope with difficult parenting situations.

## Method

### Participants

This study was part of an international collaboration (“International Investigation of Parental Burnout”; IIPB), the goal of which was to collect data on parental burnout in an Austrian sample. For further details and results of the cross-country study, please see Roskam et al. (2021). To participate, parents had to be 18 years or older and have at least one child aged 0–17 years still living at home. The Austrian study was approved by the Ethics committee of the University of Klagenfurt in February 2019.

Potential participants were recruited by means of an advertising campaign on social media that targeted family-orientated websites (i.e., parent information and support groups) as well as university-related websites (i.e., university- and student-created Facebook pages) around Austria. In addition, the study was promoted in Klagenfurt (the capital city of Carinthia), where efforts were directed towards child-related services such as Kindergartens, NGO’s and public amenities. In total, 245 eligible parents were surveyed, 88.2% of them were women, and 11.8% were men with a mean age of 36.7 years (*SD* = 8.1). Data were cleaned and checked for outliers. Parents who did not meet the eligibility criteria but still participated (i.e., youngest child was 18 or parents without children) were excluded from analysis (*n* = 5). Due to the low participation rates of fathers, only mothers’ data could be evaluated.

For the current investigation, mothers living in a committed relationship with a minimum of one child aged three years or older were included in the analyses so that dysfunctional parenting, IPV victimization, and couple dissatisfaction could be examined. This resulted in a final sample of 121 mothers with an average age of 38.9 years (*SD* = 7.5; min = 25; max = 55). The mean age of the youngest and oldest child was reported by the mother, with a mean age of 5.8 years (*SD* = 4.9; min = few months; max = 16) for the youngest child and a mean age of 9.8 years (*SD* = 6.4; min = 3; max = 30) for the oldest child. Further demographic information can be obtained in Table 1.

**Table 1:** Mothers’ Demographic Information

Variable	*M* (SD) or %
Paid profession	
Yes	81.0%
No	19.0%
Education Level **(**number of years spent in school or further education)	13.48 (2.72)
Socioeconomic background	
1 = relatively disadvantaged neighborhood	0.0%
2 = average neighborhood	65.3%
3 = relatively wealthy neighborhood	34.7%
Family type	
1 = two parent household	91.8%
2 = single parent	0.0%
3 = step family	6.6%
4 = homosexual parenthood	0.8%
5 = multigenerational household	0.8%
Number of biological children	2.04 (0.87); min = 0; max = 7
Number of children living at home	1.99 (0.78); min = 1; max = 5
Presence of child during work	
0 = never	77.7%
1 = sometimes (e.g., during school holidays)	19.0%
3 = always	3.3%
Number of hours spent with child	8.15 (3.71); min = 3; max = 24

*N* = 121. Continuous variables are reported as means, including the corresponding standard deviation. Categorical variables are reported as percentages.

### Procedure and Measures

Before participating in this study, parents were required to give informed consent and, to this end, were provided with information about the primary goal of the research project and documentation outlining the study protocol (i.e., data management and participant anonymity). First, participants were presented questions regarding demographics, such as age, gender, education level, family type, number of children, and relationship status. After completing these questions, all participants received measures regarding the constructs investigated in the current study. The order of the measures presented to the participants was the following: parents were first asked about their couple satisfaction (CSI-4), then they were presented a measure on dysfunctional parenting (PS), which was followed by a screener on physical intimate partner violence (IPV) victimization. It was decided to position the IPV screener towards the end of the survey as it was thought that the sensitive nature of the topics addressed by the questions included in this measure could potentially deter respondents from taking part. Furthermore, we paid attention to not group measures with similar topics to avoid carry-over effects.

After the IPV measure, parents were presented questionnaires from the IIPB protocol, which were shown in a fixed order to assure comparability across countries participating in the international investigation of parental burnout. As the first questionnaire of this protocol, participants were presented items on parental burnout (PBA), followed by a questionnaire on involvement in parental duties and responsibilities (PF) and a measure on gender roles (GR). Furthermore, throughout the survey, participants were presented three attention check questions (at the beginning, middle, and before the end of the survey) to assess whether they filled out the questions conscientiously. The survey lasted approximately 40 to 45 minutes. For participation in this study, parents had the chance to win one out of fifteen gift vouchers with a value of 25 euros each.

#### Parenting Scale (PS)

The German version (EFB; Naumann et al., 2010) of the PS (Arnold et al., 1993) was used to measure dysfunctional parenting strategies. Each of the 35 items were rated on a 1 to 7 Likert Scale, with higher scores indicating more dysfunctional parenting (“When my child misbehaves … I rarely use bad language or curse - I almost always use bad language”). Besides a total score, three subscales can be calculated: overreactivity, laxness, and verbosity. The PS is one of the most commonly used measures for assessing dysfunctional parental discipline in clinical and research settings (Graaf et al., 2008, Pritchett et al., 2011). Its psychometric properties (i.e., validity and reliability) have been investigated in several studies (Salari et al., 2012). Internal consistency for the total score was acceptable (*α* = 0.82).

#### Couple Satisfaction Index (CSI-4)

The German version (Treffner & Foran, 2017) of the CSI-4 (Funk & Rogge, 2007) was utilized to assess partners’ relationship dissatisfaction. One item asked about the overall happiness with the current relationship (“Please indicate the degree of happiness, all things considered, of your relationship”), which could be rated on a 7-point Likert Scale (0 = extremely unhappy to 6 = perfect). The other three items asked about positive interaction with the partner as well as the general satisfaction with the relationship (e.g., “I have a warm and comfortable relationship with my partner”) and were rated on a scale ranging from 0 to 5 (0 = not at all true to 5 = completely true). Higher scores indicate greater relationship satisfaction. The psychometric properties (i.e., reliability and validity) of the full CSI version and its shorter versions (CSI-16 and CSI-4) were satisfactory (Funk & Rogge, 2007). Internal consistency for the CSI-4 total score was excellent (*α* = 0.93).

#### Intimate Partner Violence victimization (IPV victimization)

Physical IPV was assessed using the German version of the IPV screener (Heyman et al., 2013). This measure was developed based on proposed revisions of the ICD-11 trials in a large population sample and has shown high sensitivity and specificity. Fourteen items asked about the frequency of physical violence in a relationship either as a perpetrator (“How often have you pushed, shoved or slapped your partner”) or victim (“How often has your partner pushed, shoved or slapped you”) within the last year (0 = never happened to 6 = more than 20 times in the past year). For the current study, the 7 items measuring IPV victimization were utilized. For this scale, reliability indices, as a measure of consistency, were not calculated, given that one act of physical force against the partner does not necessarily have to co-occur with another, different act of physical force. For example, one partner could have indicated slapping (or being slapped by) his/her partner several times but not biting or throwing something at the partner. As a result, these items might not be intercorrelated; however, criteria for the presence of IPV would still be fulfilled. For a similar example in the context of corporal punishment, see Lorber and Slep (2018).

#### Parental Burnout Assessment (PBA)

Parental burnout was assessed using the German translation (Prandstetter et al., 2019) of the PBA (Roskam et al., 2018). Mothers were presented with 23 items and asked to rate how often they perceived feelings related to PB (e.g., “I feel completely run down by my role as a parent”) on a 7-point Liker Scale, ranging from 0 = never to 6 = every day. Higher scores indicated higher levels of PB. The PBA includes four subscales: exhaustion in parental role, contrast with previous parental self, feelings of being fed up, and emotional distancing. Previous studies on the validity and reliability of the PBA have supported its psychometric quality (Aunola et al., 2020, Roskam et al., 2018). Cronbach’s α for the total score was excellent (*α* = 0.95).

#### Gender Roles (GR)

Mothers’ attitudes towards gender roles were assessed using the GR questionnaire (Constantin & Voicu, 2015). It includes three subscales: traditionalist, androgynist, and specialist gender roles. For purposes of the current study, only the five-item subscale on specialist gender roles (i.e., women are seen as equal to men but socialized in different tasks, such as caretaking of the household and family) was used. Mothers were presented statements regarding these gender roles and asked to indicate their amount of agreement on a Likert Scale from 1 = strongly disagree to 7 = strongly agree (e.g., “A preschool child is likely to suffer if his or her mother works”). Higher scores reflect higher levels of agreement. Internal consistency was acceptable (*α* = 0.74). The scales of the GR questionnaire are among the most commonly utilized questions in cross-cultural surveys, such as the International Social Survey Programme (ISSP) or the World Values Survey (WVS) (Constantin & Voicu, 2015, Motiejunaite & Kravchenko, 2008).

#### Involvement in Parental Function and duties scale (PF)

Mothers’ responsibility for child care was assessed using the PF. This measure was explicitly designed for the IIPB study and includes three subscales: child-rearing, basic needs, and material subsistence. The psychometrics of this measure are currently being investigated. Mothers were presented with different parental responsibilities and asked to indicate whether they took care of them on their own or shared the work with someone else (e.g., “Being present during the child(ren)’s meal”). In this study, only the 5-item subscale on child(ren)’s basic needs was used. Each item was answered on a 5-point Likert Scale (1 = Me exclusively to 5 = Someone else exclusively*)*. Lower scores indicate more responsibility for child care. Cronbach’s α was sufficient (*α* = 0.75).

It should be noted that the original PBA, GR, and PF measures were provided in English and translated to German for the current study purpose. This process involved a forward- and backward translation conducted by two doctoral students with experience in psychometrics and clinical psychology (one English- and one German native speaker) and one student assistant (German native speaker). The final translation was checked by two experts from health psychology and diagnostics (English native speakers).

### Analytic Strategy

To test the interplay between specialist gender roles, parental responsibilities, IPV victimization, couple dissatisfaction, PB, and dysfunctional parenting, structural equation modeling (SEM) was conducted in Mplus version 6.1 (Muthén & Muthén, 1998–2017). Overall, there were low rates of missing data (approximately 4.6%). However, to account for missingness and non-normality of variables, maximum likelihood estimation with robust standard errors (MLR) was used as estimator choice, utilizing full information maximum likelihood (FIML) (Enders & Bandalos, 2001). Bootstrapping analysis was conducted to obtain biased-corrected confidence intervals as recommended for mediation analysis with small sample sizes (Shrout & Bolger, 2002). To assess model fit, the following criteria were applied: an insignificant *χ*^*2*^ Model test, Comparative Fit Index (CFI) > 0.90, Tucker-Lewis Index (TLI) > 0.90, the root mean square error of approximation (RMSEA) < 0.06, and the standardized root mean square residual (SRMR) < 0.08 (Hooper et al., 2008, Hu & Bentler, 1999).

**Fig. 1:**
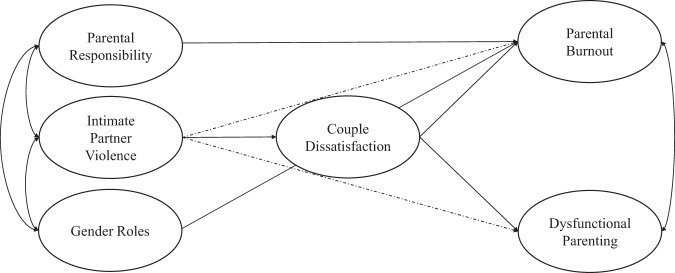
A Theoretical Model Testing for the Interplay Between the Study Variables. Note. Dotted lines indicate test for mediation

Besides the theoretically proposed model in this study (see Fig. 1), two alternative models that plausibly could fit the data were tested. These included 1) the investigation of whether IPV victimization mediates the link from mothers’ reports of couple dissatisfaction to PB as well as the link from mothers’ reports on couple dissatisfaction to dysfunctional parenting; and 2) the examination of whether couple dissatisfaction serves as a mediator of the relationship between mothers’ reports on PB and IPV victimization and mothers’ reports on dysfunctional parenting and IPV victimization.

## Results

### Descriptive Analysis

Descriptive statistics and correlations are presented in Tables 1, 2, and 3. Perceived couple dissatisfaction (CSI1, CSI2, CSI3, and CSI4), PB, and dysfunctional parenting behaviors were significantly intercorrelated. Self-reports on parental responsibilities and specialist gender roles were also significantly intercorrelated and associated with IPV victimization but not with PB. Furthermore, IPV victimization was only significantly linked to PB and couple dissatisfaction but not to dysfunctional parenting. Additionally, mothers’ reports on chronic parenting stress were significantly correlated with the number of children living in the household and the age of the youngest child but not with the hours spent with the child(ren) per day, the number of biological children as well as the age of the oldest child.

**Table 2:** Descriptive Statistics

Variable Name	Min	Max	Mean	SD	Skewness	Kurtosis
Age mother	25.00	55.00	38.90	7.46	0.27	−0.82
No. biological children	0.00	7.00	2.05	0.87	1.63**	8.09**
No. children living at home	1.00	5.00	2.00	0.78	0.77**	1.30
Hours spent with child/day	3.00	24.00	8.18	3.71	1.29**	2.02**
CSI1	1.00	6.00	3.75	1.53	−0.05	−0.89
CSI2	0.00	5.00	3.55	1.22	−0.64**	−0.13
CSI3	0.00	5.00	3.37	1.32	−0.61**	−0.62
CSI4	1.00	5.00	3.43	1.28	−0.62**	−0.66
PBA	0.00	11.10	1.96	1.87	2.14**	6.36**
IPVvict.	0.00	23.00	0.52	2.68	6.61**	47.72**
PS total	1.77	4.31	3.09	0.54	−0.23	0.04
PF Basic Needs	1.14	3.29	2.25	0.47	−0.07	−0.44
Specialist Gender Roles	0.00	28.00	9.74	6.03	0.65**	0.10

*No.* Number. **Mark skewness and kurtosis values outside the recommended range (skewness = −0.5 and 0.5; kurtosis = −2 and +2). It should be noted that the PBA usually ranges from 0 to 138, however, we adjusted the PBA scores by dividing them by 10 to match its maximum range with the other variables within the model. *CSI* Couple Satisfaction Index. *PBA* Parental Burnout Assessment. *IPV*_*vict*_*.* Intimate Partner Violence victimization screener. *PS total* Parenting Scale total score. *PF Basic Needs* Parental responsibility for basic needs of child(ren).

**Table 3:** Correlations Among Study Variables

Variables	1	2	3	4	5	6	7	8	9	10	11	12	13	14	15
1 Age mother	–														
2 No. biological children	0.15	–													
3 No. children in household	0.12	0.88**	–												
4. Age oldest child	0.70**	0.33*	0.21*	–											
5 Age youngest child	0.66*	0.03	−0.04	0.76**	–										
6 h spent with child	−0.45**	0.07	0.10	−0.37**	−0.55**	–									
7 PS	0.02	−0.08	0.04	−0.17	−0.15	0.05	–								
8 CSI1	0.10	−0.01	−0.08	0.12	0.11	−0.16	−0.22*	–							
9 CSI2	0.11	−0.03	−0.14	0.10	0.10	−0.01	−0.29**	0.72**	–						
10 CSI3	0.10	0.027	−0.03	0.11	0.10	−0.07	−0.27**	0.72**	0.84**	–					
11 CSI4	0.15	0.086	−0.03	0.17	0.13	−0.06	−0.23*	0.73**	0.85**	0.90**	–				
12 PBA	−0.09	0.091	0.19*	−0.16	−0.23*	0.12	0.28**	−0.29**	−0.32**	−0.29**	−0.32**	–			
13 IPV_vict._	−0.03	0.068	0.08	−0.05	−0.08	0.03	0.08	−0.27**	−0.30**	−0.26**	−0.28**	−0.33**	–		
14 PF basic needs	0.16	−0.13	−0.22*	0.14	0.15	−0.13	−0.05	0.16	0.13	0.16	0.12	−0.15	−0.19*	–	
15 GR specialist	−0.20*	0.07	0.15	−0.17	−0.17	0.33**	0.13	−0.03	−0.08	−0.02	−0.01	0.13	0.20*	−0.31**	–

**Correlations are significant at the 0.01 level. *Correlations are significant at the 0.05 level. *No.* Number. *PS* Parenting Scale. *CSI* Couple Satisfaction Index. *PS* Parenting Scale. *PBA* Parental Burnout. *IPV*_*vict.*_ Intimate Partner Violence victimization screener. *GR specialist* Specialist gender roles. *PF basic needs* Parental responsibility for basic needs of child(ren). No significant differences in the PS scores between parents with one or more child(ren) living in the household emerged, *t*(119) = −1.80, *p* = 0.07.

Independent *t*-tests were performed to analyze differences in mothers’ reports on PB, IPV victimization, couple dissatisfaction, parenting behaviors, specialist gender roles, and time spent with the child(ren) with respect to the profession (paid profession vs. non-paid) and the socio-economic status (average vs. wealthy neighborhood). Results indicated no significant differences between mothers with a paid profession and those without with respect to their levels of perceived PB, *t*(119) = −0.11, *p* = 0.91, IPV victimization, *t*(119) = −0.18, *p* = 0.86, couple dissatisfaction, *t*(119) = −0.21, *p* = 0.83, and dysfunctional parenting behaviors, *t*(119) = 0.40, *p* = 0.97. However, we obtained significant results for specialist gender roles, *t*(119) = −5.75, *p* < 0.001, and hours spent with the child(ren), *t*(119) = −6.60, *p* < 0.001, with mothers without a paid profession showing higher means on gender roles (*M* = 15.52) compared to those with a paid profession (*M* = 8.39). We observed a similar effect for mothers with and without a paid profession (*M* = 7.26 and *M* = 12.13) with respect to the number of hours spent with the child(ren).

Regarding SES, no significant difference between mothers from average compared to wealthy neighborhoods emerged with respect to reports of PB, *t*(119) = −0.57, *p* = 0.57, IPV victimization, *t*(119) = −0.37, *p* = 0.72, couple dissatisfaction, *t*(119) = −0.10, *p* = 0.82, dysfunctional parenting behaviors, *t*(119) = −0.80, *p* = 0.43, specialist gender roles, *t*(119) = −0.99, *p* = 0.33, and hours spent with the child(ren), *t*(119) = 1.38, *p* = 0.17.

### IPV Victimization, Couple Dissatisfaction, Dysfunctional Parenting, and Parental Burnout

**Fig. 2:**
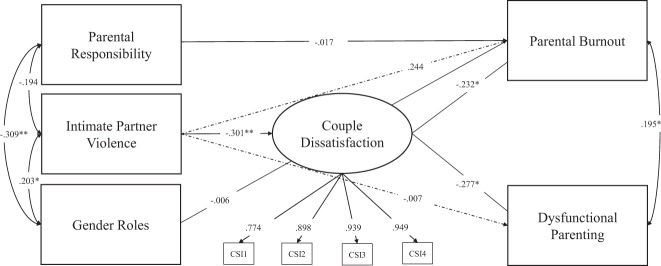
Empirical Model Testing for the Indirect Effect of Couple Dissatisfaction on Intimate Partner Violence Victimization to Parental Burnout and Intimate Partner Violence to Dysfunctional Parenting. Note. The graph contains the standardized estimates of the respective regression paths. Number of children in the household and age of the youngest child were included as control variables as they were significantly related to parental burnout. The full model included *N* = 121 mothers. Statistical significance for indirect effects is shown in Table 4

As shown in Fig. 2, SEM revealed a good model fit, *χ*^*2*^(37) = 35.51*, p* = 0.54; *CFI* = 1.00; *TLI* = 1.00; *SRMR* = 0.07; *RMSEA* = 0.00, (95% CI = 0.00–0.06) for our theoretically proposed model. In the case of the current analysis, the CFI and TLI indicate that the chi-square value is smaller than the degrees of freedom, which indicates a good model fit. Mediation analysis revealed significant indirect effects of couple dissatisfaction on the relation between IPV victimization and PB as well as the relation between IPV victimization and dysfunctional parenting (for details on direct and indirect effects, see Table 4).

**Table 4:** Test of Direct and Indirect Paths Including Intimate Partner Violence Victimization, Couple Dissatisfaction, Parental Burnout and Dysfunctional Parenting Utilizing MLR and Bootstrapping

Test for direct effects	Standardized MLR Estimate/S.E.	95% biased-corrected CI
Intimate Partner Violence Victimization → Parental Burnout	0.24 (0.21)	[−0.16; 0.53]
Intimate Partner Violence Victimization → Dysfunctional Parenting	−0.01 (0.04)	[−0.08; 0.06]
Intimate Partner Violence Victimization → Couple Dissatisfaction	−0.30 (0.08)**	[−0.42; −0.15]
Couple Dissatisfaction → Dysfunctional Parenting	−0.28 (0.10)*	[−0.42; −0.11]
Couple Dissatisfaction → Parental Burnout	−0.23 (0.10)*	[−0.40; −0.05]
Test for indirect effects		
Intimate Partner Violence → Couple Dissatisfaction → Parental Burnout	0.07 (0.03)*	[0.02; 0.14]
Intimate Partner Violence → Couple Dissatisfaction → Dysfunctional Parenting	0.08 (0.04)*	[0.03; 0.16]

*p < 0.05. **p < 0.001; *95% CI* 95 % Confidence Interval. Direct and indirect effects were tested using both, MLR and Bootstrapping.

Mothers’ self-reports on specialist gender roles and parental responsibilities were significantly intercorrelated, however, in the SEM, only specialist gender roles were significantly associated with IPV victimization in mothers. Furthermore, responsibility for child(ren)’s basic needs and specialist gender roles were also not significantly related to PB in the final model. However, couple dissatisfaction was significantly linked to both PB and dysfunctional parenting, and PB was also significantly correlated with dysfunctional parenting in the final model. Furthermore, IPV victimization was also significantly related to couple dissatisfaction but not to dysfunctional parenting behavior or PB. For detailed results of the theoretically proposed model, see Fig. 2. Additionally, results for the first alternative model tested in the current study indicated that IPV victimization did not function as a mediator of associations between couple dissatisfaction and dysfunctional parenting, nor couple dissatisfaction and PB, c′ = 0.00 [−0.02; 0.03], *p* = 0.85, c′ = −0.07 [−0.21; 0.04], *p* = 0.32, respectively. Analyses of the second alternative model showed that couple dissatisfaction was a significant mediator of the association between PB and IPV victimization, c′ = 0.06 [0.01; 0.16], *p* = 0.02; however this could not be confirmed for the association between dysfunctional parenting and IPV victimization, c′ = 0.04 [0.00; 0.13], *p* = 0.15.

## Discussion

Parental burnout (PB) is a relatively new and under-studied construct. While previous research has confirmed its negative association with parental and child mental health, to the best of our knowledge, no previous study has investigated how experiences of IPV relate to chronic parenting stress from a family-psychological perspective. Following concepts of FST and Spillover Theory, emphasizing the importance of interparental relationship quality for the parenting domain (Cox & Paley, 1997, Engfer, 1988), we tested for the mediational role of couple dissatisfaction, assuming that IPV victimization in mothers would be indirectly linked to perceived levels of PB and dysfunctional parenting behavior. In line with previous literature, we further postulated that higher levels of agreement towards specialist gender roles and more responsibility for the child(ren)’s basic needs would be intercorrelated and concurrently associated with IPV victimization and PB in mothers.

Consistent with previous results on chronic parenting stress (Mikolajczak, Raes, et al., 2018), in the present study, mothers’ self-reports on PB were significantly linked to couple dissatisfaction and negative parenting behaviors. SEM supported our main hypothesis that couple dissatisfaction mediated associations between IPV victimization and PB and IPV victimization and dysfunctional parenting. In line with our hypotheses, postulating that specialist gender roles and parental responsibilities for child(ren)’s basic needs will be intercorrelated and both related to IPV victimization and PB in the final SEM model, these assumptions could only partially be confirmed. While specialist gender roles and parental responsibilities were significantly interrelated, only specialist gender roles were associated with IPV victimization, which is in line with theories proposing power imbalances and traditional gender roles as risk factors for male to female violence (Ali & Naylor, 2013, Yllö & Bograd, 1988). These results also match with literature on the concept of “intimate terrorism” (Johnson, 1995), which emphasized coercive and controlling characteristics as key components of this type of relationship violence (Johnson et al., 2014). Although the differentiation between intimate terrorisms as opposed to situational couple violence lies beyond the scope of this study, the present findings indicate that future research could benefit from a more nuanced analysis of IPV. Such an analysis could take into account the different concepts of relationship violence and how they relate to variables at individual, interparental, and child levels, particularly in the context of PB.

Contrary to our hypothesis that specialist gender roles and overload of parental duties will make mothers more vulnerable to PB, responsibility for the child(ren)’s basic needs and mothers’ attitudes towards specialist gender roles were not significantly related to PB in the final model. While PB and IPV victimization were significantly correlated in the descriptive analyses, the path was no longer significant in the final SEM model. Also, the link from IPV victimization to dysfunctional parenting was not significant in the final model. This, however, corresponds to our correlational results (see Table 3).

As expected and in line with findings from Mikolajczak, Raes et al. (2018), couple dissatisfaction and parenting behaviors were significantly associated with PB in the SEM framework, underscoring the importance of these variables for understanding PB from a family-psychological perspective. Furthermore, our model results match with findings from studies such as the one by Brown et al. (2020), which showed that coping on the couple level was significantly related to relationship satisfaction and parenting stress in a dyadic framework. Furthermore, the authors found that couple satisfaction mediated the association between dyadic coping and reported levels of parenting stress, which emphasizes the importance of couple satisfaction as a potential protective factor for (chronic) parent stress.

Although findings from the current study also show preliminary evidence for the mediational role of mothers’ couple dissatisfaction on associations between IPV victimization and PB as well as dysfunctional parenting behaviors, these results should be interpreted with caution. Due to the cross-sectional nature of our data, no causal directionality between the study variables can be determined. Given that past literature also discussed the role of PB as a potential risk factor for child neglect (Mikolajczak, Brianda et al., 2018) and parental mental health outcomes (van Bakel et al., 2018), future longitudinal studies are needed to establish temporal associations between the constructs investigated in this paper (Maxwell et al., 2011, Maxwell & Cole, 2007), particularly with respect to open questions, such as the directionality of parent- and couple-related variables.

Moreover, all measures used in the current study were self-report questionnaires, and future research should thus consider using observational measurement approaches to minimize social desirability. For instance, dysfunctional parenting could be assessed utilizing a standardized parent-child interaction task to obtain an objective measure of parenting behaviors (Lorber et al., 2003). Additionally, concerning families with two or more children, parents were not advised which child to refer to when completing the measures on dysfunctional parenting and parental responsibilities. While there were no significant differences in dysfunctional parenting between mothers with one child and those with multiple children, as well as no significant correlations between parental responsibilities and the age of the youngest and oldest child (see Table 3), this still represents a limitation of the current study and should be taken into account in future research involving these constructs.

Furthermore, although the mean, standard deviation, and prevalence of PB in the current study were located in the lower range, our results are consistent with findings from other high-income countries, such as Italy, the Netherlands, or Sweden (Roskam et al., 2021). As previous studies have shown, families from low-and middle-income countries are more likely to be exposed to factors contributing to parenting challenges and parental stress, such as poverty, food insecurity, and community or family violence (Berger, 2005, Kieling et al., 2011). Characteristics of the current sample, such as that all mothers came from relatively wealthy or average neighborhoods and that the majority of mothers had a paid profession, may thus serve as an explanation of lower rates of PB and IPV victimization in the current study while at the same time limiting the generalizability of findings to other socioeconomic statuses and cultures.

Additionally, the relatively small sample size should be considered as a weakness of this study. Participation rates among male caregivers were too low to be statistically evaluated in the current analyses, and as such, no conclusions can be made about fathers with respect to the interplay of the variables investigated in this paper. As fathers have shown to be highly underrepresented in research on parenting (Fabiano, 2007), future studies should therefore consider a multiple informant approach, testing this model in a longitudinal dyadic framework. For instance, Roskam and Mikolajczak (2020) recently highlighted the importance of acknowledging gender differences in the context of PB. While mothers displayed higher levels of exhaustion and emotional distancing, fathers seemed to experience stronger feelings of wanting to escape and suicidal ideation when affected by PB (Roskam & Mikolajczak, 2020). These results emphasize the need for the inclusion of both parents’ data in future research to not only investigate gender differences with respect to family- and relationship-related variables but also to acknowledge the potential interdependence of parents’ perceptions and behaviors when investigating PB. Moreover, measures on children’s psychological and physical well-being should be included in future studies to examine direct or indirect effects of PB, IPV, couple dissatisfaction, and parenting behaviors on children’s mental and physical health.

Despite the above-mentioned limitations, one significant strength of this study lies in its novelty. Since previous research indicated a lack of knowledge on PB in the context of family research, the current study endeavored to bridge this gap by testing the interplay of gender roles, parental responsibilities, IPV victimization, couple dissatisfaction, PB, and dysfunctional parenting in a sample of Austrian mothers.

Connecting to the Risk and Resource Theory (Mikolajczak & Roskam, 2018), underscoring the relevance of familial risk factors for chronic parenting stress, the present study provides preliminary evidence for the importance of couples’ relationship dissatisfaction in understanding links between experiences of physical aggression and feelings of PB as well as dysfunctional parenting in mothers. On the basis of these findings, future intervention efforts may be organized to focus on preventing PB either indirectly or directly by targeting couples’ relationship quality. Indeed, couples-focused interventions appear well suited to reduce both interparental conflict and negative parenting (Gattis et al., 2008, Katz & Low, 2004). Decreasing couple conflict and violence may help to reduce parental stress (Camisasca et al., 2016, Owen et al., 2006), which in turn could minimize the risk of developing PB. The current results are also particularly relevant given the COVID-19 pandemic, which may place families at even higher risk for IPV, couple dissatisfaction, dysfunctional parenting, and PB. As rates of family violence and parenting challenges (e.g., family confinement, economic vulnerability) have significantly increased during the pandemic (Bradbury-Jones & Isham, 2020, Cluver et al., 2020), viewing these factors from a systemic perspective may lead to more effective intervention and prevention guidelines in future research.
